# Effects of a pre-workout supplement on hyperemia following leg extension resistance exercise to failure with different resistance loads

**DOI:** 10.1186/s12970-017-0195-6

**Published:** 2017-09-26

**Authors:** Jeffrey S. Martin, Petey W. Mumford, Cody T. Haun, Micheal J. Luera, Tyler W. D. Muddle, Ryan J. Colquhoun, Mary P. Feeney, Cameron S. Mackey, Paul A. Roberson, Kaelin C. Young, David D. Pascoe, Jason M. DeFreitas, Nathaniel D. M. Jenkins, Michael D. Roberts

**Affiliations:** 1 School of Kinesiology, Auburn University, Auburn, AL 36849 USA; 2 Department of Cell Biology and Physiology, Edward Via College of Osteopathic Medicine-Auburn Campus, 910 S. Donahue Drive, Auburn, AL 36832 USA; 3 School of Kinesiology, Applied Health and Recreation, Oklahoma State University, Stillwater, OK 74078 USA

**Keywords:** Reactive hyperemia, Sports supplements, Resistance exercise, Oxygenation, Blood flow, Nitric oxide, Volume load

## Abstract

**Background:**

We sought to determine if a pre-workout supplement (PWS), containing multiple ingredients thought to enhance blood flow, increases hyperemia associated with resistance training compared to placebo (PBO). Given the potential interaction with training loads/time-under-tension, we evaluated the hyperemic response at two different loads to failure.

**Methods:**

Thirty males participated in this double-blinded study. At visit 1, participants were randomly assigned to consume PWS (Reckless™) or PBO (maltodextrin and glycine) and performed four sets of leg extensions to failure at 30% or 80% of their 1-RM 45-min thereafter. 1-wk. later (visit 2), participants consumed the same supplement as before, but exercised at the alternate load. Heart rate (HR), blood pressure (BP), femoral artery blood flow, and plasma nitrate/nitrite (NOx) were assessed at baseline (BL), 45-min post-PWS/PBO consumption (PRE), and 5-min following the last set of leg extensions (POST). *Vastus lateralis* near infrared spectroscopy (NIRS) was employed during leg extension exercise. Repeated measures ANOVAs were performed with time, supplement, and load as independent variables and Bonferroni correction applied for multiple *post-hoc* comparisons. Data are reported as mean ± SD.

**Results:**

With the 30% training load compared to 80%, significantly more repetitions were performed (*p* < 0.05), but there was no difference in total volume load (*p* > 0.05). NIRS derived minimum oxygenated hemoglobin (O_2_Hb) was lower in the 80% load condition compared to 30% for all rest intervals between sets of exercise (*p* < 0.0167). HR and BP did not vary as a function of supplement or load. Femoral artery blood flow at POST was higher independent of exercise load and treatment. However, a time*supplement*load interaction was observed revealing greater femoral artery blood flow with PWS compared to PBO at POST in the 80% (+56.8%; *p* = 0.006) but not 30% load condition (+12.7%; *p* = 0.476). Plasma NOx was ~3-fold higher with PWS compared to PBO at PRE and POST (*p* < 0.001).

**Conclusions:**

Compared to PBO, the PWS consumed herein augmented hyperemia following multiple sets to failure at 80% of 1-RM, but not 30%. This specificity may be a product of interaction with local perturbations (e.g., reduced tissue oxygenation levels [minimum O_2_Hb] in the 80% load condition) and/or muscle fiber recruitment.

## Background

The phenomenon of local hyperemia in response to muscle contraction is a product of various factors including, but not limited to, metabolic signals (e.g., pCO_2_/pO_2_, lactate, K^+^, adenosine), endothelium-derived factors (e.g., nitric oxide [NO], prostacyclin, and endothelium derived hyperpolarizing factor [EDHF]), and erythrocyte ATP release [1]. With respect to metabolic signals, increased metabolic flux/demand associated with a given exercise is associated with elevations in local pCO_2_, lactate, and adenosine levels which, collectively, potentiate vasodilation through arteriole smooth muscle cell relaxation [2]. Moreover, K^+^-efflux from working skeletal muscle under conditions of high action potential frequency can hyperpolarize smooth muscle cells and promote vasodilation [1, 3]. Endothelium-derived factors that interact with vascular smooth muscle cells and likely mediate hyperemia are released in response to blood flow shear stress as well as mechanical deformation/compression of the arterioles [4, 5]. Finally, mechanical deformation and/or decreased oxygen saturation levels during exercise may elicit erythrocyte ATP release promoting vasodilation [6, 7]. The relative contribution of these vasodilatory mediators, in sum and over the time course of the response, has been difficult to quantify due to heterogeneity in metabolic perturbations throughout the skeletal muscle as well as heterogeneity in the vascular phenotype throughout the skeletal muscle arterioles [8, 9]. Regardless, it is likely that at the local tissue level, exercise-induced hyperemia involves a complex interaction of many or all of these factors.

While the formulation of ‘pre-workout’ supplements (PWS) varies markedly from product to product, many of these formulas contain a variety of ingredients, blended together, which may act to enhance blood flow in response to resistance training stimuli (i.e., exercise induced hyperemia). PWS “blends”, particularly those marketed for increased blood flow (e.g., N.O.-Xplode®, Nitraflex®, etc.), often include, but are not limited to, creatine, L-arginine, and L-citrulline. Creatine supplementation with resistance training has been shown to significantly increase peripheral blood flow with resistance training, but it is unclear if the mechanism is due to changes in NO synthesis or total body water [10]. L-arginine, which can be used to form NO in a NO synthase (NOS) catalyzed reaction [11], supplementation has been shown to increase muscle blood volume following resistance training stimuli [12]. Moreover, supplementation with L-citrulline, a precursor to L-arginine, has been shown to dose-dependently increase plasma arginine levels and NO-dependent signaling in healthy men and women [13]. Collectively, many of the blood flow potentiating ingredients including in a PWS are included in an effort to increase NO synthesis via provision of additional substrate for NOS catalyzed NO formation (i.e., feed forward).

Recently, the PWS Reckless™ (Maxium Human Performance [MHP] LLC, West Caldwell, NJ USA) was designed and includes not only the aforementioned ingredients, but also additional ingredients which may potentiate blood flow via alternative mechanisms. Indeed, the Reckless™ formula also includes the less common PWS ingredients L-norvaline, ancient peat and apple fruit extract (elevATP®), and Spectra™. L-norvaline acts as an arginase inhibitor and has been shown to augment NO production in animal models [14, 15]. Thus, L-norvaline may act synergistically with L-argininie and L-citruline by increasing bioavailability of L-arginine for NO formation. Spectra™, a “full-spectrum” antioxidant product comprised of fruit, vegetable and herb extracts, may also act in a synergistic fashion with other ingredients by reducing NO scavenging by reactive oxygen species and improving NO bioavailability [16]. Finally, ancient peat and apple fruit extract has been shown to enhance circulating ATP levels [17, 18] which may augment blood flow in a NOS independent manner [19, 20]. Indeed, although many PWS marketed to increase the blood flow response to resistance training focus on NO modulation, the contribution of NO to resistance exercise induced hyperemia is not absolute and likely partially includes ATP-sensitive potassium channels [21] which may interact with ingredient(s) found in a PWS (e.g., elevATP®).

While many studies have demonstrated the effects of individual ingredients, investigations of formulations with many potentially synergistic ingredients are sparse. Notably, many PWS formulations, including Reckless™, also include additional ingredients targeting such things as increases in perceived energy (e.g., caffeine), strength/performance (e.g., caffeine, β-alanine), and focus (e.g., whole coffee fruit extract). Considerable attention has been given to the NO potentiating effects of PWS, but, other ingredients, in theory, may accentuate factors mediating hyperemia from the exercise stimulus as a result of greater effort, training loads, etc.

To our knowledge, little is known regarding the effectiveness of PWS in different training conditions (varying time under tension, load, etc.). With varying training conditions there are likely differences in local perturbations of the factors contributing to exercise-induced hyperemia (e.g., metabolic signals, redox status, endothelium-derived factors, ATP concentration, etc.). Moreover, relative muscle activation varies markedly with high (80% of 1-repetition maximum [1-RM]) vs. low (30% of 1RM) training loads [22]. Thus, variations in training factors such as time-under-tension/training load may also interact differentially with PWS ingredients secondary to resultant differences in local perturbations and relative muscle fiber recruitment.

While the ergogenic value of improved local blood flow in response to resistance training is controversial, it may help to facilitate muscle repair [23–27]. Moreover, the post-exercise blood flow response which contributes to tissue volumizing (i.e., the “muscle pump”), is often sought by recreational and competitive resistance trainees alike. Herein, we sought to determine the effect of a multi-ingredient PWS on leg extensor resistance training-mediated hyperemia with different relative training loads. Training loads were pre-determined as low (30% of 1-RM) and high (80% of 1-RM) loads. Resistance exercise bouts included four sets to repetition failure under both conditions. Four sets to failure were selected to 1) ensure marked differences in time-under-tension between the load conditions and 2) to improve generalizability to practical settings as larger training adaptations occur with a greater number of sets [28]. We hypothesized that 1) greater time under tension would be associated with a greater exercise-induced hyperemia (femoral artery blood flow) and 2) PWS would be associated with increased circulating concentrations of NO metabolites and an accentuated exercise-induced hyperemic response. In addition, we sought to explore a potential interaction between PWS and exercise load.

## Methods

### Subjects

Thirty (*N* = 30) apparently healthy males were recruited from the local community through advertisement to participate in this double-blinded study. Inclusion criteria included being aged 18–35 years, BMI between 23 and 30 kg/m^2^, and ≥ 6 months of lower body resistance training history (i.e., resistance training legs at least once per week). Potential participants were excluded if they consumed >300 mg of caffeine per day (in beverages or supplements), consumed creatine monohydrate, beta-alanine, or pre-workout supplements in the previous two weeks, or had been regular tobacco users in the last year. To improve generalizability of the study, coffee/caffeine consumption in the prior two weeks was not an absolute exclusion criteria given that 85% of the U.S. population consumes at least 1 caffeinated beverage a day [29]. However, participants were asked to abstain from caffeine consumption (besides that included in the PWS) on the day of each visit. All procedures described herein were approved by the Auburn University Institutional Review Board and conformed to the standards set by the latest revision of the Declaration of Helsinki.

### Study design and procedures

After meeting inclusion/exclusion criteria and providing their informed consent, participants reported to the laboratory for 3 separate visits. For each visit, subjects were instructed to arrive at the same time of day in a hydrated state ≥4-h fasted and to avoid all lower body resistance exercise for at least 24-h prior. At visit #1, height and weight was measured (weigh beam scale with height rod; Detecto 439, Deteco Scale, Webb City, MO USA). Thereafter, leg extensor strength was assessed via a ‘plate-loaded’ training apparatus (Body-Solid®, GCEC340, Forest Park, IL, USA). Finally, after the leg extensor strength testing, participants were randomly assigned to either supplementation Group ‘A’ or ‘B’. Both groups were given canisters, labeled only as ‘A’ or ‘B’ for respective groups, containing a soluble powder for consumption. Participants were provided with instructions for consuming 2 scoops (~6.8 g of PWS total; maximum manufacturer recommended dose) of the supplement per day in the morning for the next 6 days. On day 7, participants reported for visit #2 (described below) and consumed the supplement in the lab. Notably, some of the ingredients included in the Reckless™ PWS generally require a loading phase for best results (e.g., creatine). Thus, although the focus of this work was to evaluate the acute effect of the PWS on reactive hyperemia responses to resistance training, one week of ‘loading’ prior to testing days was included to maximize responses.

For visit #2, participants returned to the lab and baseline (BL) venipuncture (see venipuncture section) and heart rate (HR), blood pressure (BP), and right femoral artery blood flow measures were performed at rest. Thereafter, participants consumed 2 scoops (i.e., 1 serving) of their respective supplement (‘A’ or ‘B’) and rested for 45-min. During the 45-min rest time, participants were fitted with a near infrared spectroscopy system (NIRS; Artinis Portamon, MKII, Einsteinweg 17, Netherlands) for measurement during subsequent exercise. At 45-min following ingestion of the respective supplement, pre-exercise (PRE) venipuncture and HR, BP, and femoral artery blood flow measured were again performed. This was followed by completion of 4 sets of leg extensions to failure at a load of either 30% or 80% (counter-balanced) of their pre-determined 1-RM. At exactly 5-min following leg extension exercise (POST) another blood sample was collected along with measures of HR, BP, and femoral artery blood flow. Thereafter, participants were dismissed and instructed to continue consuming their supplement in the morning each day.

Seven days following visit #2, participants reported to the laboratory for visit #3. Visit #3 was performed in exactly the same manner as visit #2 with the lone exception of the intensity of leg extension exercise performed being the intensity not performed at visit #2 (e.g. if 30% at visit #2, then 80% at visit #3 and vice versa).

#### Leg extensor strength testing (1RM determination)

At visit #1, leg extensor strength was assessed via the ‘plate-loaded’ training apparatus. Briefly, after participants were positioned on the training apparatus they completed a 10 repetition warm-up set of bilateral knee extensions with a 45lbs (20.5 kg) load followed by a 2-min rest. Thereafter, the load was increased by 25lbs (11.4 kg) and participants were instructed to perform only one repetition followed by 2-min of rest. If participants were successful with the lift, the load was increased another 25lbs followed by another 2-min of rest. This process continued until there was an unsuccessful lift and the load at the last successful lift was recorded as the 1-RM.

#### Supplementation

Investigators and participants were blinded to supplement assignments until following completion of the study. Procedurally, canisters, provided by MHP, were simply labeled as supplement ‘A’ or ‘B’. One set of the canisters contained a placebo (PBO), consisting of equal parts maltodextrin and glycine (3.4 g of each per serving herein). The other set of canisters contained a multi-ingredient PWS supplement (Reckless™; Maximum Human Performance, West Caldwell, NJ USA). Contents of the PWS and dosages consumed with a serving herein are shown in Table 1. PWS ingredients included the aforementioned blood flow potentiators (i.e., L-arginine, L-citrulline, creatine, L-norvaline, elevATP®, and Spectra™) as well as ingredients targeting improved energy (e.g., caffeine/caffeine-like [e.g., yerba mate, caffeine anhydrous, Teacrine®]), strength/performance (e.g., caffeine/caffeine-like, β-alanine), focus (e.g., Ashwaganha root and leaf [Sensori®], whole coffee fruit extract [NeuroFactor™], L-theanine), and recovery (folate). A single serving of both the PBO and PWS was two level “scoops” (~6.8 g) of the powder dissolved in 16 oz. of water. Participants ingested a serving of their respective supplement daily through the end of the entire study.

**Table 1:** Pre-workout supplement (Reckless™) ingredients and ingredient amounts consumed per serving

Ingredient(s)	Amount
Niacin (as Niacinamide)	18 mg
Folate	150 μg
Yerba Mate (leaf) (llex paraguaniensis (4:1 ext), (30% natural caffeine, 20% theophylline)	300 mg
Caffeine Anhydrous	300 mg
Ashwagandha (*Withania somnifera*) root and leaf (10% withanolides) (as Sensoril®)	126 mg
Whole coffee (*Coffea Arabica*) fruit extract (as NeuroFactor™)	100 mg
L-Theanine	100 mg
Theacrine (as TeaCrine®)	50 mg
β-alanine (as CarnoSyn®)	1600 mg
Creatine Monohydrate	1500 mg
Creatine-HCl	500 mg
Creatine-AAB (alpha amino-butyrate)	500 mg
Ancient peat and apple fruit extract (from Malus domesticus) (as elevATP®)	150 mg
L-Citrulline	1000 mg
L-Norvaline	200 mg
Spectra™ * Consisting of green coffee extract, green tea extract, broccoli sprout concentrate, onion extract, apple extract, acerola extract, camu camu concentrate, quercetin, tomato concentrate, broccoli concentrate, acai concentrate, basil concentrate, cinnamon concentrate, garlic concentrate, oregano concentrate, turmeric concentrate, carrot concentrate, elderberry concentrate, mangosteen concentrate, blackberry concentrate, blackcurrant extract, blueberry extract, chokeberry concentrate, raspberry concentrate, sweet cherry concentrate, spinach concentrate, kale concentrate, bilberry extract, brussel sprout concentrate*	100 mg
Black pepper (*Piper nigrum*) fruit extract (as Bioperine®)	5 mg

Presented are the ingredients and ingredient amounts in a single serving of the mutil-ingredient energy supplement (MIES) utilized herein (Reckless™; Maximum Human Performance, West Caldwell, NJ USA). TeaCrine® is a registered trademark and protected by Patents Pending, Serial No. 61/903,362; under exclusive global distribution by Compound Solutions Inc. Natural Alternatives International (NAI) is the owner of patents as listed on www.carnosyn.com and registered trademark CarnoSyn®. NeuroFactor(tm), elevATP™ and Spectra™ are trademarks of VDF FutureCeuticals Inc. Sensoril is a trademark of Natreon, Inc. and is protected under U.S. Patents 6,153,198 & 7,318,938. BioPerine® is a registered trademark of Sabinsa Corp.®

#### Venipuncture

Venipuncture was performed on three occasions at visits #2 and #3; at baseline, 45-min post-PWS or PBO ingestion, and 5-min following leg extensor exercise for assessment of plasma NOx (nitrate + nitrite), as described in detail below. Briefly, ~4 mL of venous blood was collected from the antecubital space in tubes containing EDTA and immediately centrifuged at 4000 g at 4 °C for 5-min. Plasma was then separated in cyrotubes and immediately placed in a − 80 °C freezer for later analysis of NOx. Plasma samples were frozen for no more than 3 months. Following deproteinization (cold-ethanol preparation), batch processing for assessment of plasma NOx was performed by ozone-based chemilluminescence using a NO analyzer (Sievers NOA 280i, Zysense, Waxhaw, NC USA). To determine NOx, samples were added to 0.1 M vanadium chloride in 1 M hydrochloric acid refluxing at 95 °C under nitrogen. The respective reduction to NO was detected by a cooled, photomultiplier tube housed in the NOA and concentrations of NOx were determined by plotting signal (mV) against a calibration plot.

#### Heart rate and blood pressure measurement

HR and BP were measured on three occasions each during visits #2 and #3; at baseline, 45-min after supplement ingestion, and 5-min post-workout. For baseline and 45-min post supplement ingestion measures, participants rested in a supine position in a quiet, temperature controlled room for 10-min prior to measurements. Post-workout measures were made at exactly 5-min following completion of the workout with the participant having ~1-2-min of supine rest time before measurement commenced. At rest, and 45-min post supplement ingestion HR, brachial systolic (SBP) and diastolic (DBP) BP measurements were made in duplicate at the brachial artery of the left arm using an automated oscillometric device (OMRON BP785, Omron Corporation, Kyoto, Japan). If consecutive SBP or DBP measures were ≥6 mmHg apart, an additional measurement was made. The two closest values were then averaged to represent BP and HR at a given time point. A single BP measurement was made at the post-exercise time point to assure consistent measurement timing of BP and femoral artery blood flow measures. Mean arterial pressure (MAP) was calculated as [PDBP +1/3(PSBP-PDBP)].

#### Femoral artery blood flow measurement

Immediately following HR and BP measurements, blood flow through the right common femoral artery was assessed using high resolution ultrasound (Logiq S7 R2 Expert; General Electric, Fairfield, CT USA) with a 3 to 12 MHz multi-frequency linear phase array transducer. The femoral artery was imaged longitudinally with the transducer placed 2–3 cm proximal to the bifurcation. Simultaneous measurement of artery diameter and blood velocity was performed using duplex mode imaging (B-mode and Doppler) and video was captured through a digital interface at 30 frames/s with real time analysis (FMD Studio, Pisa, Italy). Measurements were captured for 1 continuous min with the transducer held manually in the same position. Vessel diameters were determined frame-by-frame via automatic edge detection software (FMD Studio, Pisa, Italy) measuring the distance between the near and far wall of the intima. Blood velocity was determined via selection of a region of interest around the Doppler waveform and a trace of the velocity-time integral was used to calculate mean velocity for each cardiac cycle. Blood flow from continuous diameter and mean blood velocity measurements during ultrasonography were calculated as [Π * (diameter/2)^2^ * time average mean velocity * 60].

#### Near infrared spectroscopy (NIRS) measures

Participants wore the NIRS apparatus during the leg extensor exercise sessions for recordings during sets of exercise and the respective rest intervals in-between. Briefly, the skin over the left *vastus lateralis* was prepped (i.e., hair removal, skin abrasion) and cleaned with alcohol, and the NIRS device (optodes) was secured with an elastic bandage and flexible tape mid-way between the anterior superior iliac spine and the lateral border of the patella. Per the manufacturer instructions, a black, light-absorbing cloth was also placed over top of the NIRS device to limit the amount of ambient light detected by the device. Relative changes from baseline were measured by a continuous wavelength NIRS device that used spatially resolved spectroscopy with a sampling rate of 10 Hz. Oxyhemoglobin and deoxyhemoglobin concentration changes were calculated from light absorbance at 758 and 847 nm using the modified Lambert-Beer Law. Following data collection, the traces were smoothed using custom-written software (LabVIEW 16.0, National Instruments, Austin TX USA) to remove residual noise and movement artifact due to rhythmic muscle contractions. The primary outcomes of interest included Min and Max oxygenated hemoglobin (O_2_Hb), deoxygenated hemoglobin (HHb), difference between O_2_Hb and HHb (HbDiff), and total hemoglobin (tHb). Both minimum and maximum NIRS derived variable values were of interest to determine the extent to which each variable was reduced during a set of leg extensions and the extent to which they recovered by the end of the respective rest period(s).

#### Leg extensor exercise

After 45-min of passive rest, subjects completed four sets of as many technically-proficient repetitions as possible of bilateral leg extensions at a load of either 30% or 80% of their tested 1-RM. Each repetition was performed in cadence with a metronome set at 40 bpm. A first metronome beep initiated concentric movement, the second sound indicated that the legs should be in full extension and held there until the third sound, at the third beep the eccentric phase began, the forth sound indicated the legs should be back to the starting position and the fifth/first sound initiated the next repetition. Thus, the estimated time under tension for each repetition was approximately 4.5-s with ~1.5-s of rest between consecutive repetitions. An unsuccessful repetition, characterized by incomplete extension of the legs at the requisite tempo of the metronome, resulted in termination of the set. Subjects were given 3-min of rest between sets. Volume loads for each set were calculated as the product of repetitions and load.

### Statistical analysis

For all statistical analyses, an alpha level of *p* ≤ 0.05 was required for statistical significance. Between groups (i.e. PWS vs. PBO) comparisons for participant characteristics were evaluated using independent-tests. Three-way repeated measures ANOVAs were used to evaluate the continuous primary dependent variables associated with this study with Huynh-Feldt correction applied to hypothesis testing. Time and load were included as within-subjects factors and supplement was included as a between-subjects factor. *Post-hoc* testing of main effects and interactions were performed using t-tests (paired t-tests for exclusively within subjects factors and independent t-test for all others) with Bonferroni adjustments for multiple comparisons (α/[number of comparisons]). Grouped data are presented as mean ± standard deviation and data respective to *post-hoc* comparisons are presented as the mean difference and [95% confidence interval (lower limit, upper limit)]. All statistical analyses were performed using IBM SPSS Statistics 22 for Windows (Chicago, IL USA).

## Results

### Subject characteristics

Subject characteristics are provided in Table 2. There were no significant between group (i.e., PWS vs. PBO) differences in age, height, weight, BMI or 1-RM (*p* > 0.05).

**Table 2:** Subject Characteristics

	Overall	PWS	PBO	p-value
Age, yrs	22.1 ± 3.5	22.2 ± 4.5	22.0 ± 2.4	0.503
height, m	1.81 ± 0.07	1.82 ± 0.08	1.79 ± 0.06	0.247
body mass, kg	86.0 ± 9.5	87.3 ± 10.2	84.8 ± 9.0	0.497
BMI, kg/m^2^	26.1 ± 2.1	26.2 ± 2.2	26.1 ± 2.0	0.845
1-RM, kg	123 ± 24	127 ± 20	120 ± 28	0.448

Data are mean ± standard deviation. *P*-values are from independent t-tests (PWS vs. PBO group). BMI, body mass index; 1-RM, 1 repetition maximum

### Leg extensor exercise performance

Data regarding the number of repetitions performed and volume load for each set and load condition is presented in Table 3. As expected, there were significant main effects of load and set and a set*load interaction for number of repetitions performed (*p* < 0.001 for all). The number of reps performed during set 1, 2, 3 and 4 were all significantly greater during the 30% load compared to the 80% load (p < 0.001 for all). For volume load, a significant main effect of set (p < 0.001) and a set*load interaction (*p* = 0.002) was observed though *post-hoc* analysis revealed no significant differences in volume load between load conditions for any set (*p* > 0.0125 for all). No significant effect of treatment or treatment interactions were observed for repetitions or volume load (p > 0.05). Finally, for total volume load (sum of all four sets), there were no main effects or intensity*treatment interaction (p > 0.05). The total volume load in the 30% load condition was 5912 ± 1097 kg and 4877 ± 1904 kg for PBO and PWS, respectively. For the 80% load condition, total volume load was 5137 ± 1410 kg and 5794 ± 1894 kg for PBO and PWS, respectively.

**Table 3:** Repetitions performed and load lifted by set for leg extensor exercise to failure at 30 and 80% of 1-repetition maximum

	30%				80%			
	*Set 1*	*Set 2*	*Set 3*	*Set 4*	*Set 1*	*Set 2*	*Set 3*	*Set 4*
*Repetitions*								
Overall	66 ± 31	34 ± 13	28 ± 14	26 ± 12	20 ± 7	14 ± 4	11 ± 3	11 ± 4
PWS	58 ± 34	28 ± 11	23 ± 13	22 ± 10	22 ± 10	14 ± 4	12 ± 3	11 ± 4
PBO	73 ± 27	40 ± 13	33 ± 14	30 ± 13	18 ± 3	15 ± 3	11 ± 3	11 ± 3
*Volume load, kg*								
Overall	2306 ± 799	1209 ± 69	993 ± 419	904 ± 299	1930 ± 772	1387 ± 381	1103 ± 313	1034 ± 374
PWS	2138 ± 977	1056 ± 380	875 ± 497	808 ± 322	2153 ± 953	1389 ± 408	1170 ± 309	1081 ± 409
PBO	2463 ± 578	1353 ± 309	1103 ± 310	993 ± 254	1721 ± 502	1386 ± 364	1040 ± 314	989 ± 345

Data are mean ± standard deviation. PWS, pre-workout supplement; PBO, placebo

### Near-infrared spectroscopy (NIRS) during leg extensor exercise

**Fig. 1:**
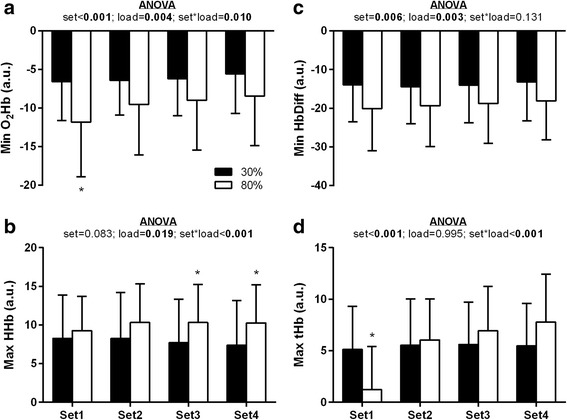
Near infrared spectroscopy variable responses during leg extensor exercise. ANOVA revealed no main effect of treatment or interaction with independent variables. Thus, **a**) minimum oxygenated hemoglobin (Min O_2_Hb), **b**) maximum deoxygenated hemoglobin (Max HHb), **c**) minimum O_2_Hb and HHb difference (Min HbDiff), and **d**) maximum total hemoglobin (Max tHb) are presented as mean values ± standard deviation for low (30% of 1RM) and high (80% of 1RM) load conditions for each set of leg extensor exercise. Relevant ANOVA *p*-values regarding set and load variables are presented within each panel. When a significant set*load interaction was found, *post-hoc* analysis with Bonferroni correction was employed for between intensity differences within each set. *, significantly different from 30% load condition (*p* < 0.0125)

For NIRS measurement during leg extensor exercise no effects (main or interactions) relative to treatment were observed. Thus, NIRS results by set and load during leg extensor exercise are illustrated in Fig. 1. During the leg extensor exercise sessions, a main effect of set and load and a set*load interaction was observed for Min O_2_Hb. Min O_2_Hb was significantly lower during the 80% load condition compared to the 30% load condition during set 1 (−5.3 [−2.8, −7.7]; *p* < 0.001) but not sets 2–4 (Fig. 1a; *p* > 0.0125 for all). For Max HHb a main effect of intensity and a set*load interaction was observed with Max HHb values being significantly higher during set 3 (+2.6 [+0.9, +4.3]; *p* = 0.004) and set 4 (+2.9 [+1.0, +4.7]; *p* = 0.004) for the 80% load condition compared to the 30% load condition (Fig. 1b). There was a main effect of set and load for Min HbDiff responses during exercise, but no set*load interaction (Fig. 1c). The Min HbDiff was significantly higher during set 4 compared to set 1 (+1.4 [+0.3, +2.4]; *p* = 0.014), set 2 (+1.3 [+0.7, +1.9]; *p* < 0.001) and set 3 (+0.8 [+0.4, +1.2]; *p* = 0.001). Moreover, Min HbDiff was significantly lower during the 80% load condition compared to the 30% load condition (−5.1 [−1.9, −8.4]; *p* = 0.003). For Max tHb, a main effect of set and set*load interaction was observed. Max tHb was significantly lower in set 1 in the 80% load condition compared to the 30% load condition (−4.0 [−1.5, −6.5]; *p* = 0.004) but not sets 2–4 (*p* > 0.0125 for all; Fig. 1d).

### Near-infrared spectroscopy (NIRS) between sets of leg extensor exercise

**Fig. 2:**
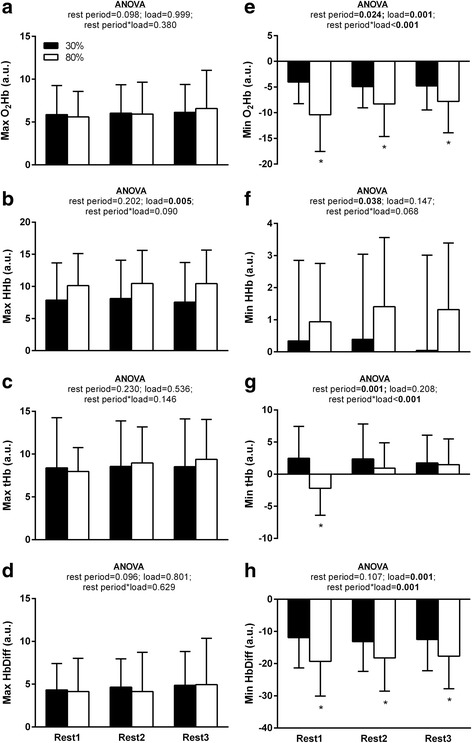
Near infrared spectroscopy (NIRS) variable responses between sets of leg extensor exercise. Maximum (Max) **a**) oxygenated hemoglobin (O_2_Hb), **b**) deoxygenated hemoglobin (HHb), **c**) total hemoglobin (tHb), **d**) O_2_Hb and HHb difference (HbDiff) and minimum (Min) **e**) O_2_Hb, **f**) HHb, **g**) tHb and **h**) HbDiff) are presented as mean values ± standard deviation for low (30% of 1RM) and high (80% of 1RM) load conditions for each set of leg extensor exercise. Relevant ANOVA *p*-values regarding set and load variables are presented within each panel. When a significant set*load interaction was found, *post-hoc* analysis with Bonferroni correction was employed for between load condition differences within each set. *, significantly different from 30% load condition (*p* < 0.0167)

NIRS results by intensity and rest periods between leg extensor exercise sets are illustrated in Fig. 2. During the rest periods between leg extensor exercise sets, no significant main effect or interactions were observed for Max O_2_Hb, Max HbDiff, or Max tHb. For Max HHb, a significant main effect of load was observed with higher values during the 80% load condition compared to the 30% load condition (+2.5 [+0.8, +4.1]; *p* = 0.005 Fig. 2b).

**Fig. 3:**
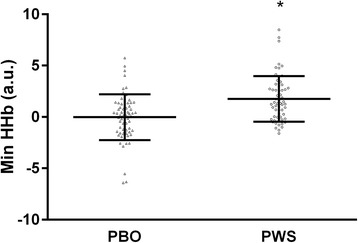
Minimum deoxygenated hemoglobin (Min HHb) across all leg extension exercise rest intervals and loads by treatment. ANOVA revealed a significant main effect of treatment (*p* < 0.05). Data are presented as individual values with solid bars indicating mean values ± standard deviation. *, significantly different from PBO (*p* < 0.05)

A main effect of rest period and load, and their interaction, was observed for MinO_2_Hb during rest periods between leg extensor exercise sets. MinO_2_Hb was significantly lower in the 80% load condition compared to the 30% load condition for rest period 1 (−6.4 [−3.9, −9.0]; *p* < 0.001), rest period 2 (−3.4 [−0.9, −6.0]; *p* = 0.011), and rest period 3 (−3.0 [−0.9, −5.1]; *p* = 0.007; Fig. 2e). For Min HHb, a main effect of rest period and treatment was observed. *Post-hoc* testing for Min HHb did not reveal significant between rest period differences (*p* > 0.0167 for all; Fig. 2f). However, Min HHb values were significantly higher with PWS treatment compared to PBO treatment (+1.8 [+0.1, +3.5]; *p* = 0.041). Fig. 3 illustrates the individual and group (i.e. treatment) mean values for Min HHb across all rest periods. For Min tHb there was a main effect of rest period and a load*rest period interaction. Min tHb was significantly lower during rest interval 1 in the 80% load condition compared to the 30% load condition (−4.1 [−1.1, −7.2]; *p* = 0.010; Fig. 2g). Finally, for Min HbDiff, a significant main effect of load and rest period*load interaction was observed with values being significantly lower during the 80% load condition compared to the 30% load condition for rest period 1 (−7.4 [−3.9, −10.9]; *p* < 0.001), rest period 2 (−5.1 [−1.8, −8.5]; *p* = 0.005) and rest period 3 (−5.2 [−2.2, −8.2]; *p* = 0.002; Fig. 2h).

### Heart rate and blood pressure

**Fig. 4:**
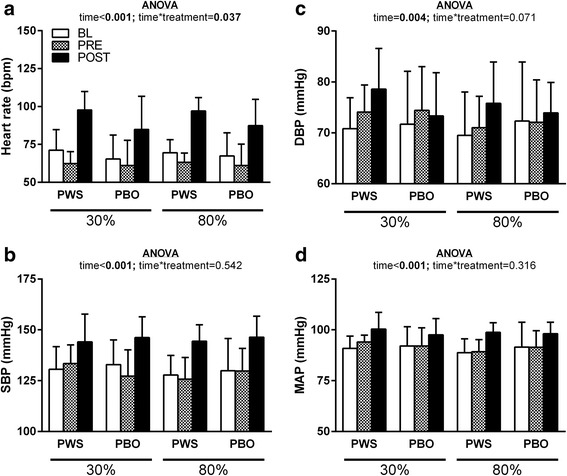
Heart rate (HR) and blood pressure (BP) values (mean ± standard devation) at baseline (BL), 45-min post ingestion of pre-workout supplement (PWS) (i.e., pre-leg extensor exercise; PRE), and 5-min post-leg extensor exercise (POST) for each load condition (i.e., 30 and 80% of 1RM) and treatment (i.e., placebo [PBO] and PWS). **a**) HR, **b**) systolic BP (SBP), **c**) diastolic BP (DBP), and **d**) mean arterial pressure (MAP). ANOVA revealed no main effects of load or its interaction with other independent variables (*p* > 0.05). Relevant ANOVA *p*-values regarding time (i.e., time point) and treatment variables are presented within each panel

Data for HR and BP measurements at BL, PRE and POST are illustrated in Fig. 4. A significant main effect of time was observed for HR, SBP, DBP and MAP. A time*treatment interaction was also observed for HR though there were no statistically significant differences observed at each time point between treatments (*p* > 0.0167 for all; Fig. 4a). Independent of treatment and load (i.e. overall mean), HR at POST was higher than at BL (+23.4 bpm [+19.0, +25.9]; *p* < 0.001) and PRE (+29.8 bpm [+25.9, +33.7]; *p* < 0.001) and HR at PRE was lower than at BL (−6.4 bpm [−4.4, −8.4]; *p* < 0.001); SBP at POST was higher than at BL (+15.0 mmHg [+11.2, +18.7]; *p* < 0.001) and PRE (+16.1 mmHg [+12.5, +19.6]; *p* < 0.001); DBP was higher at POST compared to PRE (+4.3 mmHg [+1.3, +3.8]; *p* = 0.006) and MAP was higher at POST compared to the BL (+7.9 mmHg [+5.1, +10.7]; *p* < 0.001) and PRE (+7.0 mmHg [+4.7, +9.4]; *p* < 0.001) time points.

### Femoral artery blood flow

**Fig. 5:**
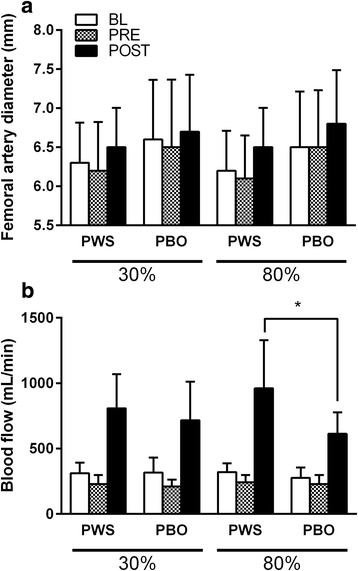
Femoral artery diameter (panel **a**) and femoral artery blood flow (panel **b**) values (mean ± standard deviation) at baseline (BL), 45-min post ingestion of pre-workout supplement (PWS) (i.e., pre-leg extensor exercise; PRE), and 5-min post-leg extensor exercise (POST) for each load condition (i.e., 30 and 80% of 1RM) and treatment (i.e., placebo [PBO] and PWS). ANOVA revealed a 3-way interaction of time, load and treatment (p < 0.05) and *post-hoc* analysis with Bonferroni correction was employed to evaluate between treatment differences at each time point for each leg extensor exercise load condition. *, significantly different from PBO at the same time point within the 80% load condition (*p* < 0.008)

Data for femoral artery diameter and femoral artery blood flow at BL, PRE and POST are illustrated in Fig. 5. A main effect of time was observed for artery diameter (*p* < 0.001). Artery diameter was significantly higher at POST compared to both BL (+0.23 mm [+0.16, +0.31]; *p* < 0.001) and PRE (+0.30 mm [+0.20, +0.39]; *p* < 0.001). For femoral artery blood flow, main effects of time and treatment as well as time*treatment, load*treatment, and time*load*treatment interactions were observed (*p*<0.05 for all). *Post-hoc* analysis revealed that 1) independent of treatment and load, femoral artery blood flow at POST was higher than at BL (+468 mL/min [+376, +560]; *p* < 0.001) and PRE (+547 mL/min [+453, +641]; *p* < 0.001) and femoral artery blood flow at PRE was lower than at BL (−79 mL/min [−59, −98]; *p* < 0.001); 2) independent of time, femoral artery blood flow was significantly higher with PWS compared to PBO in the 80% load condition (+136 mL/min [+44, +228]; *p* = 0.006), but not in the 30% load condition (+35 mL/min [−64, +134]; *p* = 0.476); and 3) blood flow was significantly higher with PWS at the POST time point compared to PBO in the 80% load condition (+348 mL/min [+124, +571]; *p* = 0.004), but not the 30% load condition (+91 mL/min [−137, +318]; *p* = 0.418).

### Nitrate and nitrite (NOx)

**Fig. 6:**
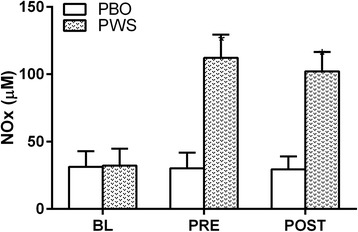
Plasma nitrate and nitrite (NOx) values (mean ± standard deviation) at baseline (BL), 45-min post ingestion of pre-workout supplement (PWS) (i.e., pre-leg extensor exercise; PRE), and 5-min post-leg extensor exercise (POST) across leg-extensor exercise load conditions (i.e., 30 and 80% of 1RM) for each treatment (i.e., placebo [PBO] and PWS). ANOVA revealed a significant time*treatment interaction (*p* < 0.05) and *post-hoc* comparisons were performed using Bonferroni correction for multiple comparisons. *, significantly different from PBO at the same time point (*p* < 0.0167)

Figure 6 illustrates plasma NOx concentrations at each measurement time point across both exercise loads. A significant main effect of time and treatment, and their interaction (*p* < 0.001 for all) was observed with significantly elevated plasma NOx concentrations in the PWS group at the PRE (+81.5 μM [+73.0, +90.0]; *p* < 0.001) and POST (+72.5 μM [+65.9, +79.1]; *p* < 0.001) time points relative to PBO.

## Discussion

The primary findings of the present study are that, 1) the magnitude of reactive hyperemia (i.e., femoral artery blood flow) following leg extensor exercise was not affected by differing estimated time under tension and/or relative loads; 2) circulating concentrations of NO metabolites (i.e., NOx) at 45-min following PWS consumption were significantly elevated and remained elevated through the end of the leg extensor exercise protocol; and 3) PWS augmented the reactive hyperemic response to leg extensor exercise at a higher relative load (80%), but not a lower relative load (30%).

PWS did not have an obvious performance effect on leg extensor exercise. The different exercise loads utilized herein resulted in markedly different numbers of repetitions performed across all sets with no significant differences in total volume loads (repetitions x load). Given that all repetitions in both exercise load conditions were performed in cadence to a metronome, estimated time under tension was also markedly different, though rest intervals between sets were the same and not standardized to exercise/work time. Thus, the primary differences between the two exercise load conditions were number of contractions, estimated time under tension, and the magnitude of force development.

During the initial set of leg extensor exercise, compared to the 30% load condition, NIRS data revealed significantly lower Max tHb and Min O_2_Hb values during the 80% load condition suggesting less perfusion relative to oxygen consumption/metabolic demand during that set. However, in the subsequent sets, Max tHb was, in essence, the same between load conditions, while Min O_2_Hb remained lower and Max HHb higher in the 80% load condition. Ultimately, the lower Max tHb values in the higher load condition during the first set could be explained by greater vascular resistance [30] and/or a reduced exercise time (~297 and 90 s for 30% and 80% load conditions, respectively) in which feedback gain from local vasodilatory mediators did not reach the same levels as in the lower load condition [31, 32]. In addition, the reduced Min O_2_Hb (and elevated Max HHb) values across all sets can likely be explained by a significantly higher work rate in the higher load condition [33].

Regarding the NIRS data in the rest periods between sets of leg extensor exercise, there appears to be a ‘carry-over’ effect from the exercise data. That is, Min O_2_Hb, HHb, tHb and HbDiff values mirror the responses observed during the exercise period. However, no differences were observed in Max O_2_Hb, HHb, tHb and HbDiff suggesting a similar ‘recovery’ over the duration of the recovery periods for both load conditions. Interestingly, we also observed a main effect of treatment on Min HHb with values being significantly higher across all rest periods with PWS. Given that PWS did not have an effect on tHb (surrogate of perfusion), we posit that the PWS effect is likely at the metabolic level. Dietary supplements increasing NOx concentrations have been shown to reduce the oxygen demand per unit of work [34–36]. Herein, the PWS acutely increased plasma NOx concentrations ~3.5-fold, a relatively large change compared to other PWS studies [37–39]. Moreover, several ingredients contained within the Reckless™ PWS (e.g., β-alanine, creatine, L-citrulline), albeit at relatively lower doses than comparative studies, have been associated with improved exercise efficiency [34, 40–42].

As noted previously, differences in NIRS parameters as a result of exercise loads in the rest periods between sets appeared to be relatively transient suggesting resolution prior to our femoral artery blood flow measurements at 5-min following the last set of leg extensor exercise. Indeed, we did not observe a time*load interaction for mean arterial blood flow data with similar values for both load conditions at the POST time point, independent of treatment. However, mean femoral artery blood flow was significantly increased at the POST time point regardless of treatment and load indicating significant exercise-induced reactive hyperemia at the time of measurement. We had hypothesized that PWS would augment the reactive hyperemic response at both exercise intensities, so it was interesting to find that the PWS had a significant effect compared to PBO in the 80% load condition, but not the 30% load condition (Fig. 6b). No differential effects of treatment or load were observed for BP or femoral artery diameters. Moreover, although there was a time*treatment interaction for HR suggesting an elevated HR with PWS at the POST time point, *post-hoc* analyses did not reveal a significant difference (*p* = 0.027) and the absolute difference in HR between PWS and PBO was actually numerically larger in the 30% load condition than the 80% load condition (Fig. 4a). Thus, hemodynamics and/or femoral artery diameter do not appear to explain the effect of PWS on mean femoral artery blood flow in the 80% load condition but not 30% load condition.

Exercise-induced reactive hyperemia involves a complex interaction of many factors (i.e., mechanical, metabolic, endothelium-derived, and erythrocyte-derived). Further obfuscating interpretation of the responses observed herein are the interaction of these factors with unique exercise loads, estimated time-under tension, force development, and PWS ingredients. Indeed, there are a plethora of ingredients included in the PWS utilized herein that could affect the reactive hyperemic response, but the specific effect of PWS on mean femoral artery blood flow post exercise in the 80% load condition but not 30% load condition is intriguing. If PWS ingredient(s)-mediated increases in local concentrations of metabolites (e.g., adenosine) were major players independent of local factors, a similar effect would be expected across both exercise loads. One notable difference observed herein was the significantly lower Min O_2_Hb and Min HbDiff values observed during the rest periods between sets of leg extensor exercise. As noted previously, it is likely that these differences ‘carry-over’ from the exercise itself and is resolved over the time course of the 3-min rest period as evidenced by similar Max O_2_Hb and HbDiff values during rest. Though transient, a period of reduced oxygenation in the local tissues could interact with circulating metabolites from the PWS and have a cumulative effect with multiple sets of exercise. Indeed, a major stimulus for nitrate/nitrite conversion to NO is hypoxia [43] which could contribute to the reactive hyperemia response. However, this notion is not supported by the Max tHb data observed during and between sets of exercise (no treatment*load interaction).

Though beyond the scope of this study, we propose two possibilities for consideration as explanatory mechanisms for the intensity specific effect of PWS. First, in the 80% load condition, the greater resistance loads and subsequent force development with leg extensor contraction may be associated with increased muscle fiber recruitment [44]. With a relatively higher proportion of active muscle fibers, there may also be increased capillary network recruitment [1] potentiating delivery of PWS ingredients and metabolites to a greater area of vascular tissue. Moreover, there is likely a differential proportion of fiber types recruited between the two loads which could interact with PWS ingredients. Indeed, there is marked heterogeneity in vascular control mechanisms as a function of fiber type [45, 46] and fiber type/region specific effects of dietary supplements (e.g., nitrate) is not novel [47, 48]. Secondly, it is possible that with the significantly greater number of repetitions and exercise time, there are higher local levels of vasodilatory metabolites and the ability of PWS to further augment the response is limited (i.e., diminishing returns).

### Limitations

Notably, the PWS administered in the current study included a relatively high dose of caffeine (390 mg) and we did not include a matched, caffeine only group. However, previous work suggests that caffeine may actually attenuate limb blood flow during dynamic leg exercise [49]. Regardless, the Reckless™ PWS includes several ingredients, including caffeine, which may have impacted the findings. The intention of this study was to evaluate a PWS as a whole, though this makes it difficult to discern if the results were attributable to any one ingredient in particular or any synergy between ingredients. Notably, the relative dose delivered with a serving for some of the blood flow potentiating ingredients is less than found in more focused supplements/PWS (e.g. only 500 mg of L-citrulline) which may suggest that there is some additive effect of multiple ingredients and/or synergy between ingredients. However, in the context of the current study, the mechanisms underlying the reported effects cannot be delineated. More research is warranted on the synergistic and isolated effects of the ingredients found in PWS. In addition, our study included a cohort with a relatively high degree of experience in training (at least 6 consecutive months of lower body resistance training prior to the study), and further work should determine the effects of this PWS with varying resistance training ages. Finally, we did not monitor dietary habits throughout the study beyond the requirement of reporting for visits ≥4-h fasted. Thus, the influence of dietary behaviors in the days leading up to visits cannot be excluded.

## Conclusions

In summary, we report that four consecutive sets of leg extensor exercise to failure at 30% and 80% of 1RM elicits a similar post-exercise reactive hyperemic response with greater hemoglobin deoxygenation during exercise that resolves quickly during rest (<1 min). The PWS utilized herein did not affect exercise performance or NIRS variables during exercise, though Min HHb was found to be higher with PWS. Finally, reactive hyperemia was significantly increased compared to PBO post-exercise but only following the 80% exercise load condition. Local metabolic perturbations and fiber-type specific effects of the PWS should continue to be explored in an effort to further define which situations and athletes may benefit from its use.

## Abbreviations

1-RM: 1-repetition maximum
BP: Blood pressure
DBP: Diastolic blood pressure
EDHF: Endothelium derived hyperpolarizing factor
HbDiff: Difference between O_2_Hb and HHb
HHb: Deoxygenated hemoglobin
HR: Heart rate
MAP: Mean arterial pressure
Max: Maximum
Min: Minimum
NIRS: Near infrared spectroscopy
NO: Nitric oxide
NOx: Nitrite + nitrate
O_2_Hb: Oxygenated hemoglobin
PBO: Placebo
PWS: Pre-workout supplement
SBP: Systolic blood pressure
tHb: Total hemoglobin
